# Phylogeography of the California sheephead, *Semicossyphus pulcher*: the role of deep reefs as stepping stones and pathways to antitropicality

**DOI:** 10.1002/ece3.840

**Published:** 2013-10-21

**Authors:** Marloes Poortvliet, Gary C Longo, Kimberly Selkoe, Paul H Barber, Crow White, Jennifer E Caselle, Alejandro Perez-Matus, Steven D Gaines, Giacomo Bernardi

**Affiliations:** 1 Department of Ecology and Evolutionary Biology, University of California Santa CruzSanta Cruz, California, 95076; 2 Department of Marine Benthic Ecology and Evolution, Centre for Ecological and Evolutionary Studies, University of GroningenNijenborgh 7, 9747 AG, Groningen, The Netherlands; 3 Marine Science Institute, University of California Santa BarbaraSanta Barbara, California, 93106; 4 Hawai'i Institute of Marine Biology, University of Hawai'iKane'ohe, Hawaii, 96744; 5 Department of Ecology and Evolutionary Biology and the Institute of the Environment and Sustainability, University of California Los Angeles621 Charles E. Young Dr. South, Los Angeles, California, 90095; 6 Biological Sciences Department, California Polytechnic State UniversitySan Luis Obispo, California, 93407; 7 Subtidal Ecology Laboratory & Center for Marine Conservation, Pontificia Universidad Católica de Chile, Estación Costera de Investigaciones MarinasCasilla 114-D, Santiago, Las Cruces, Chile; 8 Bren School of Environmental Science and Management, University of CaliforniaSanta Barbara, California, 93106

**Keywords:** Antitropicality, microsatellites, *Semicossyphus*, sheephead wrasse, speciation, stepping stones

## Abstract

In the past decade, the study of dispersal of marine organisms has shifted from focusing predominantly on the larval stage to a recent interest in adult movement. Antitropical distributions provide a unique system to assess vagility and dispersal. In this study, we have focused on an antitropical wrasse genus, *Semicossyphus,* which includes the California sheephead, *S. pulcher*, and Darwin's sheephead, *S. darwini*. Using a phylogenetic approach based on mitochondrial and nuclear markers, and a population genetic approach based on mitochondrial control region sequences and 10 microsatellite loci, we compared the phylogenetic relationships of these two species, as well as the population genetic characteristics within *S. pulcher*. While *S. pulcher* and *S. darwini* are found in the temperate eastern Pacific regions of the northern and southern hemispheres, respectively, their genetic divergence was very small (estimated to have occurred between 200 and 600 kya). Within *S. pulcher,* genetic structuring was generally weak, especially along mainland California, but showed weak differentiation between Sea of Cortez and California, and between mainland California and Channel Islands. We highlight the congruence of weak genetic differentiation both within and between species and discuss possible causes for maintenance of high gene flow. In particular, we argue that deep and cooler water refugia are used as stepping stones to connect distant populations, resulting in low levels of genetic differentiation.

## Introduction

In marine fishes, population structuring at large scales is generally weak due to high effective population sizes and/or high migration rates. Similarly, speciation in the sea is thought to be counteracted by high gene flow enabled by dispersive larval forms and a rarity of strong physical barriers to dispersal and intermixing (Rocha and Bowen 2008; Puebla 2009; Bernardi 2013). Attempts at predicting population structure and gene flow among populations of marine fishes based on a number of variables, in particular the pelagic larval duration (PLD) of a given species, mostly resulted in contradictory findings (Waples 1987; Doherty et al. 1995; Shulman and Bermingham 1995; Riginos and Victor 2001; Selkoe and Toonen 2011). While correlations have been tenuous, the methods used to test these predictors have been compromised by the inherent constraints of the metrics and methods used rather than a necessarily weak relationship in nature (Weersing and Toonen 2009; Faurby and Barber 2012).

For marine organisms, dispersal was long thought to be principally achieved via a pelagic larval stage, and although larval dispersal likely plays an important role in shaping genetic patterns, evidence accumulated over the past decade indicates that local retention, in particular for fishes, is more important than once thought (Jones et al. 1999; Swearer et al. 1999; Almany et al. 2007; Saenz-Agudelo et al. 2009a; Beldade et al. 2012; Bernardi et al. 2012; Berumen et al. 2012). With the primacy of larval dispersal diminishing, the roles of ecological characteristics and dispersal of adult stages in shaping genetic population structure has in turn, taken a more important place (Schinske et al. 2010; Luiz et al. 2012). Systems where adult dispersal is likely to play a determining role are therefore important to assess. The case of antitropical distributions, for example, has long been puzzling to biogeographers and marine ecologists. For these taxa, which are present at high latitudes but absent from the intertropical regions, several scenarios of dispersal and vicariance have been proposed (Lindberg 1991). While several cases of antitropicality have been described, it has been argued that the tropical eastern pacific (TEP) is a region where tropical submergence (where deeper cooler water is found below the warm surface water) is likely to have played an important role by allowing fish to traverse the equator via the short, steep continental shelf in the eastern Pacific (Hubbs 1952; Lindberg 1991). Indeed, studies on fish species have shown a genetic link between populations in the Southern and Northern Hemisphere in the TEP (Stepien and Rosenblatt 1996; Bowen and Grant 1997). In this study, we assessed the potential for deep-water stepping stones to genetically connect *Semicossyphus* populations.

*Semicossyphus* is an antitropical fish genus in the family Labridae (Wrasses). Wrasses include a large number of predominantly coral reef species (approximately 600 species), with a basal tribe, the Hypsigenyines, that includes the temperate genus *Semicossyphus*, and its close relatives, the genera *Bodianus* and *Clepticus,* which are mostly found on coral reefs (Westneat and Alfaro 2005; Beldade et al. 2009). *Semicossyphus* includes only three species, the Asian sheephead (*S. reticulatus*)*,* Darwin's sheephead (*S. darwini*), and the California sheephead (*S. pulcher*). The Asian sheephead is found in Japan, Korea, and China (Masuda et al. 1984; Froese and Pauly 2000). Darwin's sheephead, one of the few fish species Charles Darwin collected in the Galapagos Islands (Pauly 2004), is found in deeper cooler waters of the southern and western Galapagos Islands, and coastal areas of Ecuador, Peru, and Chile (Allen and Robertson 1994; Grove and Lavenberg 1997). The California sheephead is found from Monterey Bay, California, to the northern Sea of Cortez, Mexico, including the California Channel islands and the isolated Guadalupe Island, Mexico. *Semicossyphus pulcher* was originally described as a disjunct species, where individuals are found in the northern Sea of Cortez and the northwestern Pacific coast of the Baja California Peninsula but absent from the southern Sea of Cortez and Baja California (Miller and Lea 1972; Present 1987). However, this species does occur (albeit rarely) as far south as Cabo San Lucas (the southern tip of Baja California) (Bernardi et al. 2003) therefore exhibiting a continuous range from Monterey Bay to the northern Sea of Cortez. This occurs most likely via deeper water where food resources and more homogeneous habitat is conducive to adult *S. pulcher* dispersal (Bernardi et al. 2003).

**Figure 1:**
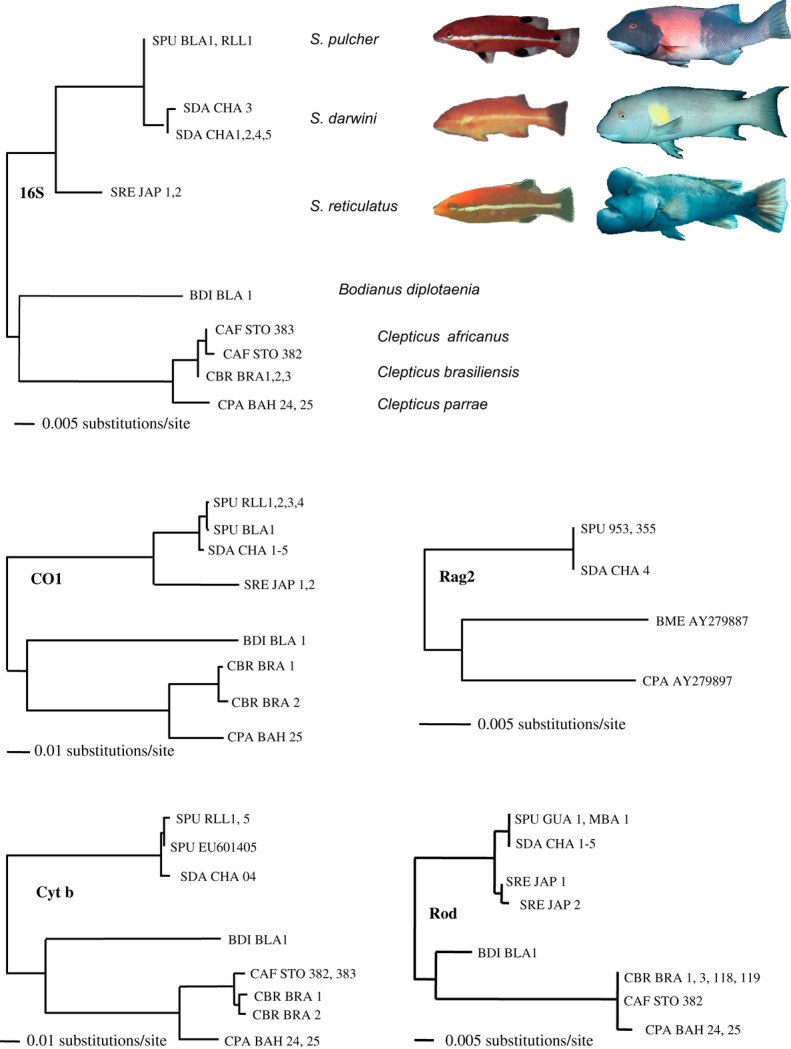
Phylogenetic relationships of the genus *Semicossyphus* based on three mitochondrial (16SrRNA, 16S; Cytochrome oxydase 1, CO1; Cytochrome b, Cytb) and two nuclear (Recombination activation factor 2, Rag2; Rhodopsin, Rod) markers. All three *Semicossyphus* species were used (*S. pulcher, S. darwini, S. reticulatus*). The two closest genera, *Bodianus* (*B. diplotaenia*), and *Clepticus* (*C. africanus, C. parrae, C. brasiliensis*) were used as outgroups. Pictures of juvenile (left) and terminal phase adult (right) *Semicossyphus* are shown to emphasise the similarity among juveniles of all three species and adult *S. pulcher* and *S. darwini*.

Sheephead are reef fish that feed mostly on benthic invertebrates such as sea urchins, gastropods, and octopus, which are resources that are found in both shallow and deeper waters, thus permitting sheephead to roam between shallow and deeper habitats (Hamilton et al. 2011). They are protogynous hermaphrodites (like most wrasses, Kazancioğlu and Alonzo 2010), with juvenile and Initial Phase (IP, female) forms looking very similar in the three species, while the Terminal Phase (TP, male) appears to be similar, except for coloration, in *S. pulcher* and *S. darwini*, but looks very different in *S. reticulatus* (Fig. 1). *Semicossyphus* are broadcast spawners and consequently produce pelagic larvae that remain in the water column for approximately 30 days, thus allowing, at least theoretically, for long-distance dispersal and high gene flow (Warner 1975; Cowen 1985; Victor 1986; Siegel et al. 2003; Andrews and Anderson 2004; Caselle et al. 2011; Hamilton et al. 2011).

The goal of this study was to assess phylogeographic patterns in the California sheephead, *Semicossyphus pulcher* and relate these patterns to its antitropical sister taxon, *S. darwini*. We used a phylogenetic approach using DNA sequences of three mitochondrial and two nuclear markers from all three *Semicossyphus* species and representatives of the two closest genera, *Bodianus* and *Clepticus* as outgroups. We used a population genetic approach using mitochondrial DNA sequences of the hypervariable control region and 10 microsatellite markers on individuals of *S. pulcher* collected across the entire range of the species, from the Monterey bay to the northern Sea of Cortez, including the California Channel Islands and Guadalupe Island.

## Materials and Methods

### Collection of samples and DNA extraction

Samples from 499 *S. pulcher* were collected from 20 locations spanning the entire range of the species, from Monterey Bay, California, to the Sea of Cortez, Mexico, including all major representative offshore islands (Table 1). Samples from five *S. darwini* were collected from Chile, and samples from two *S. reticulatus* from Japan (Table 1). Samples of the outgroup species *Clepticus africanus*, *C. braziliensis*, *C. parrae,* and *Bodianus diplotaenia* were collected from Sao Tomé, Brazil, the Bahamas, and Mexico, respectively (Table 1). DNA was extracted following a standard chloroform protocol (Sambrook et al. 1989).

**Table 1:** Sampling of *Semicossyphus, Clepticus,* and *Bodianus*. Columns correspond to collection localities, locality codes, and sample numbers for mitochondrial sequences and microsatellite analysis

Species sampling locality	Code	mtDNA	Microsats

*Semicossyphus pulcher* (SPU)	California Sheephead		
USA			
California Mainland			
Monterey bay	MOB	1	2
Palos Verdes	PVE	3	54
Point Loma	PTL		50
California Channel Islands			
San Miguel	SMI	19	
Santa Rosa Island	SRI		33
Santa Cruz Island	CRU		43
Santa Catalina Island	CAT	20	40
San Nicolas Island	SNI	18	46
San Clemente Island	SCL	20	38
Mexico Baja California, Islands			
Isla San Martin	ISM		38
Isla Cedros	CED	20	
Isla Guadalupe	GUA	13	35
Baja California, Pacific Coast			
Bahia Tortugas	BTO	25	48
Bahia Asuncion	ASU	2	2
Punta Canoas	CAN	18	
Lopez Mateos	LOM		43
Sea of Cortez			
Puerto Peñasco	PPE	4	4
Bahia de Los Angeles	BLA	5	10
Bahia San Francisquito	SFR	5	11
Los Frailes	LFR	2	2
Total	SOC	16	27

*Semicossyphus darwini* (SDA)	Darwin's sheephead		
Chile			
Caleta Chanaral	CHA	5	5

*Semicossyphus reticulatus* (SRE)	Asian sheephead	
Japan			
Iyo, Ehime	SRE	2	
Outgroups			
*Clepticus parrae* (CPA)	Creole wrasse		
Bahamas	BAH	2	
*Clepticus braziliensis* (CBR)	Brazil's Creole wrasse		
Brazil	BRA	3	
*Clepticus africanus* (CAF)	African Creole wrasse		
Sao Tome	STO	2	
*Bodianus diplotaneia* (BDI)	Mexican hogfish		
Mexico			
Bahia de Los Angeles	BLA	1	

### Phylogenetics

#### PCR amplification and sequencing

Mitochondrial cytochrome b (CYB), cytochrome oxidase I (CO1), and 16SrRNA (16S) were amplified for a subset of *S. pulcher* samples (six samples from four locations) and for all samples from the other species via PCR using primers VF2T1 and VR1dT1, 16SAR and 16SBR, and GLUDG-L and CB3H, respectively (Kocher et al. 1989; Palumbi 1996; Ivanova et al. 2007). For the same subset of samples, amplification of the nuclear RAG2 was performed using the primers RAG2F1 and RAG2R3 (Lovejoy 2000). Amplification of the nuclear rhodopsin marker (Rod) followed published nested amplification protocols (Sevilla et al. 2007), with RHO30F and RHO 319R for the first set of primers and Rho F2x and RhoR4n for the second set of primers.

All amplifications were performed in 13 μl reactions containing 0.5 μl of DNA, 0.625 μl of each primer (forward–reverse) and 11.25 ml of Thermo scientific 1.1 × PCR master mix (2.5 mmol/L MgCl2). After an initial denaturation of 1 to 3 min, 30–35 cycles at 94°C for 45 s, followed by 45 s at an annealing temperature of 52–56°C and 60 s at 72°C were conducted, followed by a final extension of 3 min at 72°C. After purification following the manufacturer's protocol (ABI, Perkin-Elmer, Foster City, CA), sequencing was performed with the primers used in the PCR amplification on an ABI 3100 automated sequencer (Applied Biosystems, Foster City, CA) at University of California Berkeley. The putative nature of each sequence was confirmed by BLASTN search. In the case of the nuclear markers, heterozygous individuals were scored using IUPAC ambiguity codes.

#### Phylogenetic analysis

Sequences were trimmed and aligned using the MAFFT (Katoh et al. 2002) routine implemented in Geneious 5.0 (Biomatters, Auckland, New Zealand). For CYB, CO1, 16S, RAG2, and Rod genes, jModeltest 2 (Guindon and Gascuel 2003; Darriba et al. 2012) was used to determine the substitution model that best fit the data based on the corrected Akaike Information Criterion. Maximum-likelihood analyses of each of these genes were performed in GARLI 2.0 (Zwickl 2006), with priors set to fit the evolutionary model suggested by jModeltest, but allowing the parameters to be recalculated during the run. Each of five independent runs was automatically terminated after 10,000 generations without improvement in topology. The support was evaluated with 100 bootstrap replicates.

Bayesian phylogenetic analyses of these same genes were run in MrBayes 3.1 (Huelsenbeck and Ronquist 2001; Ronquist and Huelsenbeck 2003) setting priors to fit the evolutionary model suggested by jModeltest but allowing the parameters to be recalculated during the run.

### Population genetics

#### PCR amplification and genotyping

The hypervariable mitochondrial control region was amplified for a subset of 175 *S. pulcher* samples from 15 locations (Table 1) and all *S. darwini* samples using the PCR primers CRA and CRE (Lee et al. 1995). A total of ten microsatellite loci were amplified for all 499 collected *S. pulcher* samples following published protocols (Poortvliet et al. 2009). Scoring of peaks was performed manually using GENEMAPPER 3.7 (Applied Biosystems). Deviations from Hardy–Weinberg equilibrium and presence of null alleles and linkage disequilibrium were estimated using ARLEQUIN 3.11 (Excoffier et al. 2005) and MICRO-CHECKER 2.2.3 (Van Oosterhout et al. 2004).

#### Population genetic analysis

A haplotype network based on mitochondrial control region sequences of *S. pulcher* samples only (175 samples) was generated in R using HaploNet in the APE 3.0-9 package (Paradis et al. 2004) combined with pie diagrams of haplotype frequencies obtained with APE and ARLEQUIN. Population genetic parameters (Fst and Φst) were calculated with ARLEQUIN, and values of Dst and Jost's D were calculated using GENODIVE (Meirmans and Van Tienderen 2004). Analyses of Molecular Variance (AMOVA) (Excoffier et al. 1992) were computed using the ARLEQUIN package.

To explore and decompose the genetic variability of microsatellite loci into gene pools without providing prior information on the geographic origin of the samples, a Bayesian clustering approach implemented in STRUCTURE 2.2 was used (Pritchard et al. 2000). The program simultaneously defines clusters and assigns individual multilocus genotypes to the defined clusters. Allele frequencies were presumed uncorrelated, and null alleles were coded as recessive to take into account the presence of null alleles in the dataset (Falush et al. 2007). The most likely number of clusters in the dataset was identified based on 10 runs using the Evanno method and visualised in STRUCTURE HARVESTER (Pritchard et al. 2000; Evanno et al. 2005; Earl and VonHoldt 2012). In addition, GENODIVE's K-means clustering was run for number of clusters (K) from 1 to N-2 using AMOVA-based simulated annealing with 50,000 steps and 20 repeats. Cluster membership was examined to determine whether adjacent sampling sites clustered together, illuminating where genetic breaks between regions might exist. Because F_st_ estimators can be insensitive when gene flow and allelic diversity are high, we also used the program SAShA (Kelly et al. 2010) to detect geographically restricted alleles and test for panmixia. Population structure in control region sequences and microsatellite genotypes was evaluated using an Analysis of Molecular Variance (AMOVA) implemented in ARLEQUIN. Several alternative groupings (California *versus* Sea of Cortez, California Channel Islands *versus* all other sampling locations and Southern Mexican islands *versus* all other sampling locations) were considered.

## Results

### Phylogenetic reconstructions

All phylogenetic reconstructions showed *S. pulcher* as a very closely related sister species to *S. darwini*, with *S. reticulatus* being distantly related to these two species (Fig. 1), regardless of marker or reconstruction method used. The sequence divergence between *S. reticulatus* and the other two species varied between 6.1% (CO1) and 4.0% (16S). The sequence divergence between *S. pulcher* and *S. darwini* was 0% for the nuclear markers (i.e., no differences at the RAG2 and Rod loci) and less than 1% for the mitochondrial markers (0.57%, 0.61%, and 0.86% for CO1, 16S, and CYB, respectively). Considering a universal substitution rate of 1.5 to 2.5% per million year in fish cytochrome b sequences (Meyer 1994), and a substitution rate of 1.2% per million year based on 19 trans-Isthmian geminate species of fish CO1 sequences (Bermingham et al. 1997; Marko 2002), the divergence time between California sheephead and Darwin sheephead was estimated at approximately 344–573 kya for cytochrome b and 475 kya for CO1.

#### Mitochondrial control region sequences

**Figure 2:**
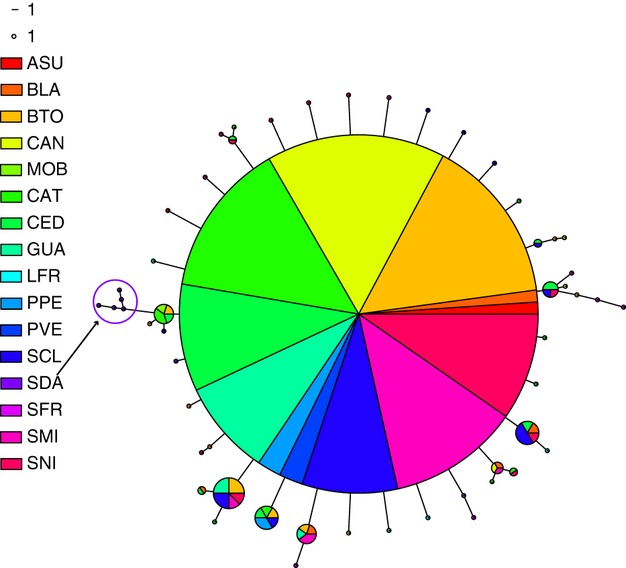
Haplotype network of *Semicossyphus pulcher* and *S. darwini* based on the mitochondrial control region (D-loop). Populations are color-coded, the size of the pies are proportional to their corresponding haplotype frequency. Population codes are given in Table 1 and Figure 3.

Sample numbers, number of haplotypes, haplotype diversity, and nucleotide diversity are given in Table 2. We obtained two sequences for *S. reticulatus,* five sequences of *S. darwini,* and 175 sequences of *S. pulcher*. As for the other molecular markers, *S. pulcher* and *S. darwini* were closely related, while *S. reticulatus* was very distantly related to the two sister species. Sequence divergence between *S. reticulatus* and *S. pulcher + S. darwini* was 27.5%. All five *S. darwini* individuals had different haplotypes from each other and none of these five haplotypes were shared with *S. pulcher* (Fig. 2). Samples of *S. darwini* grouped together in a monophyletic assemblage due to five point mutations (3 fixed, 2 nearly fixed) that separated *S. darwini* from *S. pulcher* (corresponding to a sequence divergence of 2.1%). Considering a substitution rate of 10% per million years for fish control regions (Domingues et al. 2005, 2006; Drew and Barber 2012), the divergence of *S. darwini* and *S. pulcher* based on control region sequences was estimated at approximately 210 kya.

**Table 2:** Characteristics of the mitochondrial control region in *Semicossyphus pulcher* and *S. darwini*. Locality codes are given in Table 1

Locality	n	Number of haplotypes	Haplotype diversity (standard deviation)	Nucleotide diversity (standard deviation)
MOB	1	1	1.0000 (0.0000)	0.0000 (0.0000)
PVE	3	2	0.6667 (0.3143)	0.0017 (0.0021)
SMI	19	8	0.6725 (0.1190)	0.0037 (0.0026)
CAT	20	7	0.5842 (0.1270)	0.0027 (0.0021)
SNI	18	10	0.7647 (0.1079)	0.0034 (0.0025)
SCL	20	10	0.8316 (0.0751)	0.0032 (0.0023)
CED	20	11	0.8053 (0.0903)	0.0037 (0.0026)
GUA	13	5	0.6282 (0.1431)	0.0034 (0.0026)
BTO	25	11	0.6933 (0.1034)	0.0139 (0.0024)
ASU	2	2	1.0000 (0.5000)	0.0127 (0.0139)
CAN	18	4	0.3137 (0.1376)	0.0020 (0.0017)
PPE	4	2	0.6667 (0.2401)	0.0017 (0.0019)
BLA	5	5	1.0000 (0.1265)	0.0051 (0.0040)
SFR	5	5	1.0000 (0.1265)	0.0122 (0.0083)
LFR	2	2	1.0000 (0.5000)	0.0101 (0.0113)
SDA	5	5	1.0000 (0.1265)	0.0061 (0.0046)

#### Microsatellite analyses

We analyzed microsatellites for 504 individuals. We were able to obtain microsatellite scores for all 10 loci for 499 *S. pulcher* and five *S. darwini* individuals. Specific characteristics of the microsatellite data used here are provided in Table 3. Loci were neither out of HWE nor in Linkage Disequilibrium.

As expected, all approaches separated the two species, *S. pulcher* and *S. darwini*, in two genetic clusters based on microsatellite data. The *S. darwini* samples showed 12 private alleles that are absent in *S. pulcher*, while the two most differentiated *S. pulcher* populations, SRI and SMI (northern Channel Islands), had only 2 private alleles (Table 3).

**Table 3:** Microsatellite characteristics for *Semicossyphus pulcher* and *S. darwini*. Locality codes are given in Table 1

Locality (sample #)		A4	A7	A109	C7	D2	D101	D106	D113	D118	D120	Private alleles
MOB 2	N_a_	1	3	4	3	1	1	4	1	3	4	0
	H_obs_	N/A	0.5	1	1	N/A	N/A	1	N/A	1	1	
	H_exp_	N/A	0.83	1	0.83	N/A	N/A	1	N/A	0.83	1	
PVE 54	N_a_	4	10	15	6	6	7	6	3	7	15	1
	H_obs_	0.61	0.91	0.74	0.72	0.41	0.59	0.61	0.59	0.65	0.89	
	H_exp_	0.54	0.85	0.77	0.64	0.41	0.64	0.72	0.51	0.69	0.91	
PTL 50	N_a_	3	8	12	6	4	8	6	3	9	14	1
	H_obs_	0.68	0.92	0.8	0.74	0.4	0.56	0.6	0.48	0.61	0.82	
	H_exp_	0.6	0.86	0.77	0.67	0.38	0.55	0.62	0.5	0.64	0.89	
SRI 33	N_a_	3	10	11	5	4	6	5	3	10	12	2
	H_obs_	0.58	0.94	0.82	0.5	0.48	0.61	0.45	0.64	0.7	0.85	
	H_exp_	0.53	0.84	0.82	0.6	0.45	0.62	0.6	0.52	0.65	0.88	
CRU 43	N_a_	3	10	13	5	5	7	5	3	11	14	1
	H_obs_	0.58	0.88	0.72	0.58	0.51	0.61	0.56	0.49	0.72	0.98	
	H_exp_	0.6	0.84	0.83	0.6	0.42	0.55	0.65	0.5	0.64	0.9	
CAT 40	N_a_	3	8	10	5	5	6	6	3	6	14	0
	H_obs_	0.55	0.82	0.73	0.6	0.4	0.67	0.52	0.54	0.77	0.98	
	H_exp_	0.58	0.84	0.79	0.62	0.41	0.64	0.68	0.52	0.67	0.89	
SNI 46	N_a_	3	10	10	6	5	8	5	3	11	14	1
	H_obs_	0.53	0.83	0.78	0.52	0.52	0.67	0.5	0.5	0.74	0.93	
	H_exp_	0.56	0.83	0.79	0.57	0.43	0.62	0.61	0.5	0.72	0.89	
SCL 38	N_a_	3	9	8	6	5	7	5	3	8	14	1
	H_obs_	0.7	0.84	0.71	0.55	0.57	0.53	0.79	0.74	0.78	0.87	
	H_exp_	0.59	0.83	0.73	0.5	0.53	0.59	0.72	0.54	0.67	0.91	
ISM 38	N_a_	3	11	10	5	4	7	6	2	7	14	2
	H_obs_	0.53	0.84	0.78	0.6	0.38	0.4	0.6	0.27	0.54	0.84	
	H_exp_	0.52	0.88	0.78	0.63	0.39	0.47	0.65	0.48	0.58	0.89	
GUA 35	N_a_	3	9	10	5	4	6	5	3	7	15	1
	H_obs_	0.54	0.83	0.8	0.66	0.24	0.76	0.71	0.32	0.69	0.89	
	H_exp_	0.57	0.86	0.78	0.65	0.32	0.65	0.67	0.46	0.7	0.9	
BTO 48	N_a_	3	10	10	6	5	6	6	3	10	14	0
	H_obs_	0.42	0.81	0.9	0.64	0.44	0.51	0.53	0.5	0.64	0.94	
	H_exp_	0.57	0.86	0.81	0.68	0.38	0.53	0.68	0.49	0.64	0.91	
ASU 2	N_a_	2	3	3	3	1	2	2	1	3	4	0
	H_obs_	1	0.5	1	1	N/A	0.5	0.5	N/A	0.5	1	
	H_exp_	0.67	0.83	0.83	0.83	N/A	0.5	0.5	N/A	0.83	1	
LOM 43	N_a_	4	9	10	6	4	7	5	3	9	15	1
	H_obs_	0.6	0.98	0.74	0.5	0.35	0.62	0.51	0.5	0.61	0.83	
	H_exp_	0.54	0.83	0.74	0.57	0.33	0.64	0.68	0.53	0.66	0.9	
SOC 27	N_a_	3	9	10	4	5	7	5	3	5	13	0
	H_obs_	0.59	0.7	0.78	0.38	0.44	0.58	0.44	0.48	0.59	0.74*	
	H_exp_	0.61	0.84	0.74	0.61	0.4	0.6	0.59	0.58	0.58	0.88	
SDA 5	N_a_	1	3	5	3	2	6	4	2	3	8	12
	H_obs_	N/A	0.2*	0.5	0.2*	0.2	0.6	0.6	0.2	0.6	1	
	H_exp_	N/A	0.69	0.86	0.73	0.2	0.87	0.73	0.2	0.64	0.96	

N_a_= number of alleles; H_obs_= observed heterozygosities; H_exp_= expected heterozygosities of microsatellite loci per population.

* Indicates significant deviation from Hardy Weinberg Equilibrium after Bonferroni-type corrections.

N/A indicates monomorphic, did not test.

Microsattlite loci names are given in the first row, number of private alleles are given in the right column.

### Population genetics

#### Mitochondrial control region sequences

**Figure 3:**
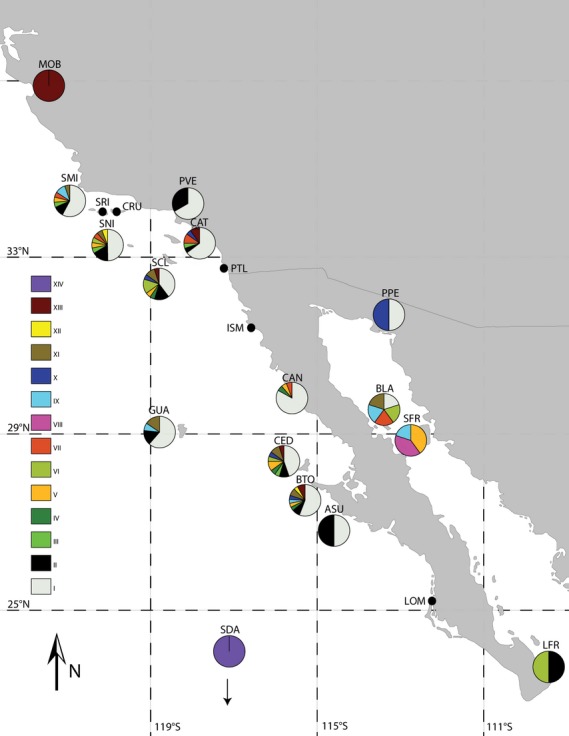
Sampling locations of California sheephead, *Semicossyphus pulcher*, and mitochondrial control region haplotypes. The most common haplotype is represented in grey, private haplotypes (only found in a given population) are represented in black. The remaining 13 haplotypes are color-coded and shown on Figure S1 as an overlay of the haplotype network of Figure 2. Solid black dots indicate additional sampling locations for microsatellites. Monterey Bay, MOB; San Miguel Island, SMI; Santa Rosa Island, SRI; Santa Cruz Island, CRU; San Nicolas Island, SNI; Santa Catalina Island, CAT; San Clemente Island, SCL; Palos Verdes, PVE; Point Loma, PTL; Isla San Martin, ISM, Punta Canoas, CAN; Isla Guadalupe, GUA; Isla Cedros, CED; Bahia Tortuga, BTO; Bahia Asuncion, ASU; Lopez Mateos, LOM; Los Frailes, LFR; San Francisquito, SFR; Bahia de Los Angeles, BLA; Puerto Peñasco, PPE.

Sample numbers, number of haplotypes, haplotype diversity, and nucleotide diversity are given in Table 2. One predominant *S. pulcher* haplotype was found in 93 individuals, from which other haplotypes or groups of haplotypes stemmed (Fig. 2). The haplotype network did not present any obvious geographic pattern; however, haplotype frequencies did show geographic patterns that could be discerned visually when placed on a map (Fig. 3). Indeed, while most locales did include the most common (grey) haplotype, ten of 15 sites had unique (black) haplotypes.

Because *S. pulcher* has traditionally been considered a Sea of Cortez disjunct species (Present 1987; Bernardi et al. 2003), we first performed an Analysis of Molecular Variance (AMOVA) by separating California and Sea of Cortez populations into two separate groups. In this case, 9.1% of the total variance could be attributed to this partition, which was statistically significant (Φ_ct_ = 0.091, *P* = 0.018). We then clustered the California Channel Islands as a separate group, because these islands have also been considered a place where genetic differences may arise (e.g., Bernardi 2000, 2005). There, only 1.1% of the total variance could be assigned to this grouping; yet, this value was still statistically significant ((Φ_ct_ = 0.011, *P* = 0.024). Finally, we considered the southern Mexican islands of Cedros and Guadalupe as another group, as recruitment dynamics at Guadalupe Island have been suggested as potentially being linked with major oceanographic shifts (Cowen 1985). In this case, however, the variance attributed to this grouping was zero (Φ_ct_ = −0.87) and was not statistically significant (*P* = 0.11). Within California samples, no pairwise comparisons of Φ_st_ or F_st_ performed in ARLEQUIN were found to be statistically significant and neither were measures of Jost's D using GENODIVE.

#### Microsatellite analyses

**Figure 4:**
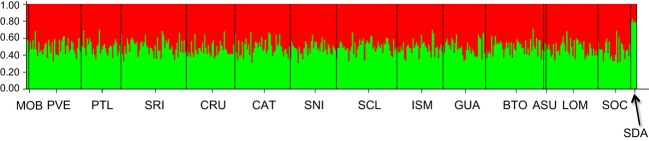
Bayesian population assignment test based on 10 microsatellites loci. Highest likelihood was found when data were partitioned in two clusters (K = 2) represented in green and red. Each vertical line represents one individual and its assignment likelihood to belong to one of the cluster (Y scale) is shown by the color. Black vertical lines represent the limit between predefined groups (populations). Population codes are given in Table 1 and Figure 1.

Because only 5 *Semicossyphus darwini* were sampled, these were not included in the population analyses based on microsatellites. AMOVA analyses of *S. pulcher* microsatellite data did not result in any significant population structure. When we separated California and Sea of Cortez populations into two groups, we found that only 0.02% of the total variance could be attributed to this partition, a result that was also not statistically significant. Clustering the California Channel Islands or the Mexican offshore islands into separate groups also yielded nonsignificant differences, with 0.12% and 0.06% of the variance assignable to these partitions, respectively. Results from STRUCTURE were consistent with the AMOVA (Fig. 4), in showing a lack of population structure of *S. pulcher* within California and so was the GENODIVE's K-means clustering analysis. The lack of concordance between mitochondrial and microsatellite results, however, is not entirely unusual and has repeatedly been observed before (DiBattista et al. 2012).

## Discussion

In marine systems, assessing population structure and diversity is complicated by the dispersive (i.e., pelagic) larval stage of most benthic organisms, a life stage that is difficult to fully assess (Paulay and Meyer 2006). Dispersal along the eastern Pacific Coast of South and North America for the sand crab *Emerita analoga* was ascribed to its long larval phase (Dawson et al. 2011). Similarly, two of three pelagic fishes tested (Stepien and Rosenblatt 1996), as well as sardines (Bowen and Grant 1997), show strong genetic connectivity between Southern and Northern Hemispheres via the coastal Tropical Eastern Pacific. In the case of the sardines, where antitropical populations diverged very recently, some haplotypes were even shared between Chile and California (Bowen and Grant 1997). Here, a very similar pattern of antitropical dispersal was shown for *Semicossyphus darwini* and *S. pulcher,* two species that may have diverged as recently as 200–600 kya. Currently, there is no known suitable habitat that intervenes their respective distributions to provide a means for stepping stone gene flow via adult migrations. The species' 1-month pelagic larval duration is unlikely to enable regular larval exchange across the tropics. Nevertheless, the phylogenetic similarity of the species suggests the potential for occasional high dispersal across the tropics, perhaps during extreme storm events. Our analysis of the population genetic structure of *S. pulcher* also suggests high dispersal potential, providing one example consistent with the idea that gene flow levels within a species may correlate with levels between species.

### Population structure and speciation in marine organisms

In general, marine organisms have large effective population sizes and have traditionally been considered mobile or at least with large dispersal potential. This is particularly true at the larval stage, where larvae are carried away from reefs by oceanographic currents. This view, that predicts shallow population structure, was challenged in the late 1990s when fish larvae were found to be retained (self-recruitment) at much higher rates than expected (Jones et al. 1999; Swearer et al. 1999). Indeed, later empirical studies have shown that a significant portion of the larval pool recruits close to the parental habitat and display behaviors that challenge the notion that they are passive dispersers (Jones et al. 2005; Gerlach et al. 2007; Planes et al. 2009; Saenz-Agudelo et al. 2009b; Beldade et al. 2012; Bernardi et al. 2012; Berumen et al. 2012). Thus, combining oceanographic factors together with larval behavior is an approach that is likely to better reflect the mechanisms of dispersal in marine species (Selkoe et al. 2010; White et al. 2010; Alberto et al. 2011; Selkoe and Toonen 2011). In addition, the ecological characteristics of adults, a factor traditionally neglected in attempts to predict population structure in marine organisms, have regained importance (Schinske et al. 2010; Luiz et al. 2012). In turn, such ecological factor may play an important population genetic role over long-time scales that would influence speciation mechanisms. Within this context, the goal of our study was to determine whether speciation-level patterns (macroevolution) matched population-level patterns (microevolution). As discussed above, dispersal across the tropics in sister species of sheephead indicates that occasional long-distance dispersal is likely in these species. Population structure analysis of the California sheephead, *S. pulcher*, is consistent with this result, with a near absence of any population structure across a geographic area that covers approximately 2,500 km of coastline from Monterey, California, to the northern Sea of Cortez.

### Dispersal capabilities and antitropical connections

Several attempts have been made at identifying the factors that influence population structure in marine organisms. While it is clear that in extreme cases, major oceanographic or physical barriers play an overriding role in structuring populations of entire biotas (Bernardi et al. 2003; Lessios and Robertson 2009; Toonen et al. 2011; Von der Heyden et al. 2011), other, more moderate situations may not be so simple. Indeed, predicting population structure has been a challenge. Here, we argue that the ecological and evolutionary mechanisms responsible for the shallow population structure observed in *Semicossyphus pulcher* are similar to the factors responsible for the low genetic separation between *S. pulcher* and its sister species *S. darwini*. We assume here that some key life history, evolutionary, and ecological factors combined with abiotic factors such as historical and oceanographic features, must play an important role.

As noted above, *S. pulcher* has been considered a disjunct species, with continuous populations in California and the west coast of Baja California, and an isolated disjunct population in the northern Sea of Cortez (Present 1987; Thomson et al. 2000), yet careful field and archival examination showed that this species is found continuously along the Baja California peninsula, albeit in deeper water, where it probably feeds on invertebrates, escapes ecological competition from shallow tropical species, and does not experience warm surface temperatures (Bernardi et al. 2003). In fact, *S. pulcher* is only seen in the shallow waters of the northern Sea of Cortez during the cold winter months and is absent during the warm summer months, when it presumably migrates to deeper, colder waters (G. Bernardi. pers. obs.). The physical connection of continuous populations via deeper waters is therefore a likely conduit to genetic connectivity in this species, resulting in shallow population structure.

The lack of large genetic separation between *S. pulcher* and *S. darwini*, two species that display antitropical distributions, may be due to several potential factors. Stepping stones as a mode of distant connectivity have been evoked for a long time (Kimura 1953; Kimura and Weiss 1964; Hellberg et al. 2002; Purcell et al. 2006; Rocha and Bowen 2008; White et al. 2010) and recently were explicitly tested using oceanographic models in a marine system (Crandall et al. 2012). Similarly to the situation described for *S. pulcher*, deep-water refugia between the distribution ranges of *S. pulcher* and *S. darwini* may have acted as stepping stones in the past. Indeed, oceanographic models of such habitats were analyzed and locations of deep-water refugia in the Tropical Eastern Pacific were predicted (Graham et al. 2007; Santelices 2007). When predictive models were tested in the field, deep-water kelp beds were found by scuba divers, and more relevant to our study, *S. darwini* individuals were observed there (Graham et al. 2007). It is therefore conceivable that gene flow between antitropical populations remained active until recently, most likely via deep-water refugia, resulting in small genetic divergence between these species in mitochondrial markers and no observed differences in the nuclear markers. In addition, the genetic divergence between *S. darwini* and *S. pulcher*, estimated to have occurred approximately 344–573 kya, encompasses the time of glaciating periods (300–455 kya). During these times, the water temperature in the tropical region was lowered, further increasing the potential for temperate fishes to breach that boundary (Chiang and Bitz 2005). The presence of fixed differences in the mitochondrial marker and several private alleles in the microsatellite markers suggests that gene flow between these species has currently stopped.

### Adaptation, Fisheries and Conservation

Sheephead are large fishes that are targeted by both commercial and sport fishermen in Chile, California, and Asia (Hamilton et al. 2007; Selkoe et al. 2007; Godoy et al. 2010; Caselle et al. 2011), thus identifying structured populations would inform managers to help conserve different stocks. Our data show a lack of genetic structure for *S. pulcher* populations in California, suggesting either a genuine panmictic scenario, or structure that is difficult to identify. While we do not know whether California sheephead are panmictic, some significant metrics (e.g., between the Sea of Cortez and the California coast, AMOVA, φ_ct_= 0.091, *P* = 0.018) suggest that structure may be present. In other systems, such as hake or cod, population structure that was not uncovered in early surveys was shown to be present when more powerful molecular markers, such as SNPs, were used, and discrete stocks and fundamental population patterns were revealed (Moen et al. 2008; Milano et al. 2011). Those results, often associated with genes under selection, are indicative of local adaptation, a factor that may ultimately be responsible for the evolution of Sea of Cortez populations and the separation between *S. darwini* and *S. pulcher*.

While our study only examines a single system, it provides a rare example where macroevolution mirrors microevolution. The ability of *S. pulcher* to achieve high gene flow, which reduces population structuring within the species, is also the most likely explanation for the very low divergence between sister species *S. darwini* and *S. pulcher*. Lack of large physical distances between suitable habitats likely enables stepping stone dispersal that counteracts divergence due to local adaptation. Despite the appearance of great physical distance between the ranges of *S. darwini* and *S. pulcher*, the presence of deep-water habitat may facilitate occasional exchange. The marine environments of Chile and California have often been compared due to their very similar characteristics, with cold currents (Humboldt and California, respectively), high productivity, similar fish assemblages, and kelp forests (Stepien 1990; Boyle and Horn 2006; Perez-Matus et al. 2012). In that respect, the *Semicossyphus* system offers a unique opportunity to understand which factors are most influential in shaping the structure of marine populations.

## Supporting Information

**Figure S1.** Haplotype network of *Semicossyphus pulcher* and *S. darwini* based on the mitochondrial control region (D-loop), as shown in Figure 2, but where each haplotype is described with a different color. The most common haplotype is in grey. Haplotypes that are unique to a population are in black. This haplotype network was used to generate the colors presented in Figure 3.
