# Supplementary figures and images for: Phylogeography of the California sheephead, Semicossyphus pulcher: the role of deep reefs as stepping stones and pathways to antitropicality
Source: Ecol Evol. 2013 Oct 21;3(13):4558–71. doi: 10.1002/ece3.840 (PMC3856754; doi:10.1002/ece3.840)

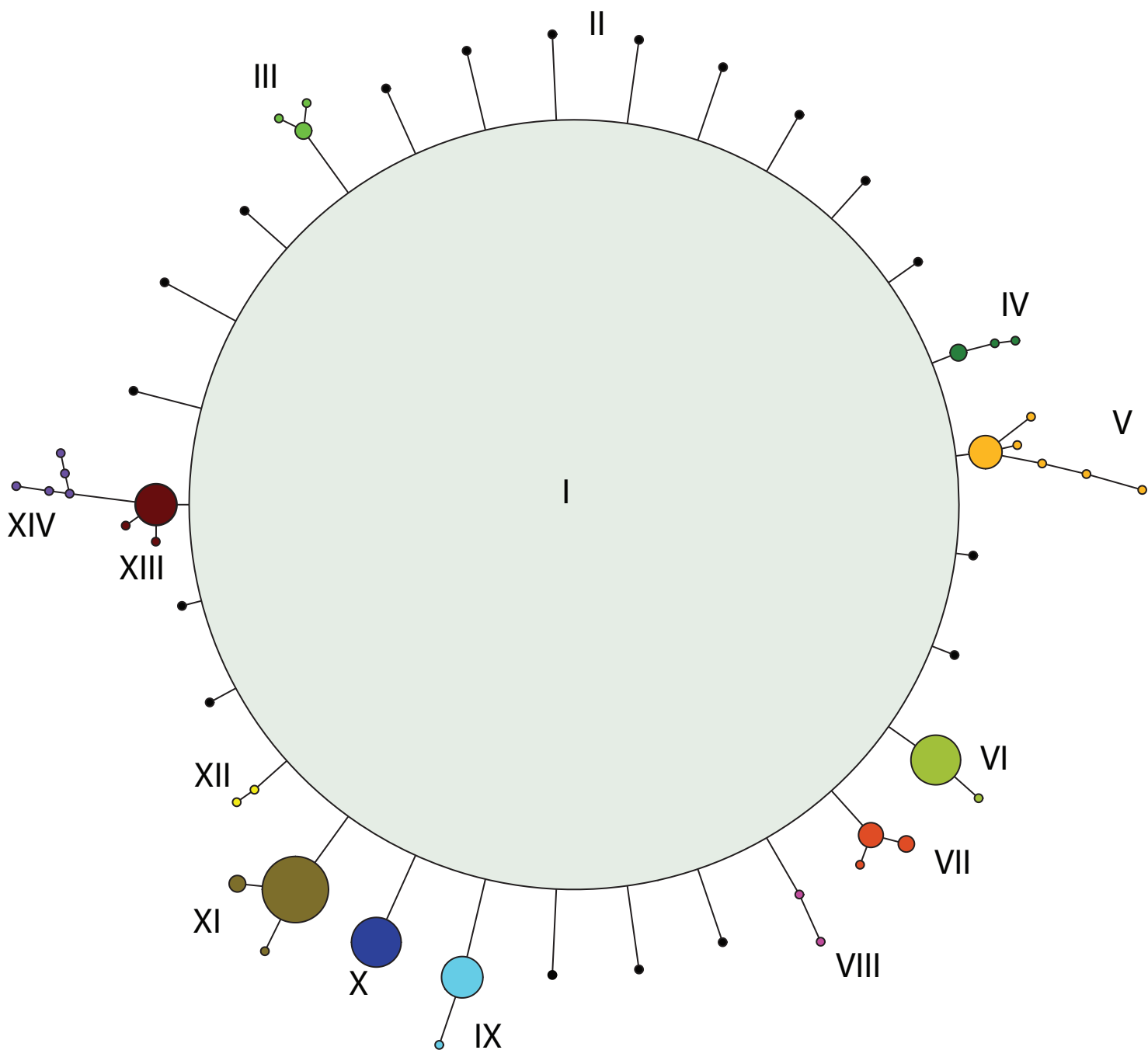

Supplement: Supplementary file 1 [file ece30003-4558-SD1.pdf]
